# Melatonin Improves Endoplasmic Reticulum Stress-Mediated IRE1α Pathway in Zücker Diabetic Fatty Rat

**DOI:** 10.3390/ph14030232

**Published:** 2021-03-08

**Authors:** Samira Aouichat, Miguel Navarro-Alarcon, Pablo Alarcón-Guijo, Diego Salagre, Marwa Ncir, Lazhar Zourgui, Ahmad Agil

**Affiliations:** 1 Department of Pharmacology, Biohealth Institute and Neurosciences Institute, School of Medicine, University of Granada, 18016 Granada, Spain; samira_aouichat@outlook.fr (S.A.); pabloang30@correo.ugr.es (P.A.-G.); dsalagres@correo.ugr.es (D.S.); 2 Team of Cellular and Molecular Physiopathology, Faculty of Biological Sciences, University of Sciences and Technology Houari Boumediene, El Alia, Algiers 16111, Algeria; 3 Department of Nutrition and Bromatology, School of Pharmacy, University of Granada, 18071 Granada, Spain; nalarcon@ugr.es; 4 Bioactive Molecule Valorization Research Unit, Higher Institute of Applied Biology of Medenine, University of Gabes, Gabes 4119, Tunisia; nsirrmarwaa@yahoo.fr (M.N.); lazhar.zourgui@gmail.com (L.Z.); 5 Biosanitary Research Institute of Granada (ibs. GRANADA), University Hospital of Granada, 18016 Granada, Spain

**Keywords:** melatonin, endoplasmic reticulum stress, diabesity, kidney

## Abstract

Obesity and diabetes are linked to an increased prevalence of kidney disease. Endoplasmic reticulum stress has recently gained growing importance in the pathogenesis of obesity and diabetes-related kidney disease. Melatonin, is an important anti-obesogenic natural bioactive compound. Previously, our research group showed that the renoprotective effect of melatonin administration was associated with restoring mitochondrial fission/fusion balance and function in a rat model of diabesity-induced kidney injury. This study was carried out to further investigate whether melatonin could suppress renal endoplasmic reticulum (ER) stress response and the downstream unfolded protein response activation under obese and diabetic conditions. Zücker diabetic fatty (ZDF) rats and lean littermates (ZL) were orally supplemented either with melatonin (10 mg/kg body weight (BW)/day) (M–ZDF and M–ZL) or vehicle (C–ZDF and C–ZL) for 17 weeks. Western blot analysis of ER stress-related markers and renal morphology were assessed. Compared to C–ZL rats, higher ER stress response associated with impaired renal morphology was observed in C–ZDF rats. Melatonin supplementation alleviated renal ER stress response in ZDF rats, by decreasing glucose-regulated protein 78 (GRP78), phosphoinositol-requiring enzyme1α (IRE1α), and ATF6 levels but had no effect on phospho–protein kinase RNA–like endoplasmic reticulum kinase (PERK) level. In addition, melatonin supplementation also restrained the ER stress-mediated apoptotic pathway, as indicated by decreased pro-apoptotic proteins phospho–c–jun amino terminal kinase (JNK), Bax, and cleaved caspase-3, as well as by upregulation of B cell lymphoma (Bcl)-2 protein. These improvements were associated with renal structural recovery. Taken together, our findings revealed that melatonin play a renoprotective role, at least in part, by suppressing ER stress and related pro-apoptotic IRE1α/JNK signaling pathway.

## 1. Introduction

Chronic metabolic diseases, particularly obesity and type 2 diabetes mellitus (T2DM), are a major health problem worldwide, and kidney disease associated to them is an important complication that is emerging as a major cause of morbidity and mortality [1]. Despite great progress in the control of obesity and T2DM, effective clinical therapy of obesity and diabetes-related kidney injury is still limited, emphasizing the urgent necessity for the development of new therapeutic strategies that target novel signaling pathways.

It is well accepted that obese and diabetic kidney microenvironments play a pivotal role in the pathogenesis of chronic kidney disease, through a mechanism involving the endoplasmic reticulum (ER), the intracellular organelle responsible for synthesis, folding, and maturation of protein, and for Ca^+2^ homeostasis modulation and lipids or steroids synthesis [2,3,4]. Pathophysiological states that increase the demand for protein folding or that disrupt normal folding processes result in accumulation of misfolded proteins in the ER and cause ER stress [3]. At the initial stage of ER stress, a protective process termed unfolded protein response (UPR) is initiated in the ER, which is mediated by three transmembrane sensors, including protein kinase RNA–like endoplasmic reticulum kinase (PERK), activated transcription factor 6 (ATF–6) and inositol-requiring enzyme1α (IRE1α) [5]. Under non-stress conditions, the proteins are bound to the ER chaperone protein glucose-regulated protein 78 kDa (GRP78), and thus remain inactive. Upon accumulation of unfolded proteins in the ER, GRP78 becomes dissociated from these transducers proteins, and UPR cascade is activated after dimerization and autophosphorylation of PERK and IRE1α, and regulated intramembrane proteolysis of ATF6. This response induced by UPR triggers the reduction in global protein synthesis, the degradation of misfolded or unfolded proteins, and the increase of protein chaperones synthesis, especially GRP78. When the adaptive ER stress fails to restore cell homeostasis, this response can also trigger an apoptotic pathway to remove the damaged cells [2]. One or more pro-apoptotic signaling pathways are known to contribute to cell death under prolonged or chronic activation of the three UPR pathways. According to the literature, UPR-mediated pro-apoptotic signaling is divided between that mediated by IRE1α and that mediated by CCAAT/enhancer-binding protein homologous protein (CHOP). Although CHOP can be induced by all three ER stress sensors, it is most strongly induced by activation of PERK. IRE1α initiates ER stress-associated apoptosis by recruiting tumor necrosis factor receptor-associated factor 2 (TRAF2), leading to the activation of the c–jun amino terminal kinase (JNK) pathway of apoptosis, which suppresses the expression of B cell lymphoma (Bcl-2) and induces that of Bcl–2-associated x–protein (Bax). Another mechanism of ER stress-associated apoptosis is via upregulation of Ca^2+^-mediated signaling, where ER stress leads to the release of Ca^2+^ from ER to mitochondria, resulting in the activation of the caspase family for apoptosis [6].

Accumulating evidence indicates that ER stress is pathogenic in various kidney diseases [7]. The link between chronic ER stress and renal damage was established by the presence of ER stress markers in both renal glomeruli and tubular interstitium of animal models of both obesity and diabetes [8,9,10,11,12] and in diabetic patients [13]. Obese and diabetic kidney microenvironment (e.g., lipotoxicity, glucotoxicity, and proteinuria) has been postulated to induce several conditions that impose an ER stress response and UPR pathway activation, leading to renal cells death [9,13,14,15]. Hence, therapeutic strategies targeting ER stress and its downstream signaling might have the potential to provide a powerful tool in an attempt to prevent renal injury in obesity and diabetes.

Melatonin (N-acetyl-5-methoxytryptamine) is a highly lipophilic molecule that can easily cross the cell membrane in many organs and organelles [16]. It is an indoleamine synthesized and secreted from the pineal gland and is mainly responsible for controlling the circadian cycle [17]. Melatonin is also produced extrapineally by many peripheral tissues, including kidney, owning to the expression of the two key enzymes involved in its synthesis (arylalkylamine-N-acetyltransferase (AA-NAT) and hydroxyindole-O-methyltransferase (HIOMT)) [18,19]. Exogenous melatonin is widely used to remedy sleep disorders and jet-lag, either as a dietary supplement or as a drug, in many countries in Europe and USA [20,21]. Apart from this, melatonin was shown to possess remarkable anti-obesity and metabolic effects, as well as robust cell-protective properties, including anti-inflammatory and anti-apoptotic properties [22,23,24]. The effectiveness of melatonin to reduce kidney injury and dysfunction caused by various pathological conditions, such as obesity and diabetes, has been widely investigated, and various mechanisms have been reported to underlie its beneficial effects, including anti-oxidative stress, anti-inflammatory, and anti-apoptotic mechanisms [16,25,26,27,28,29,30,31,32,33,34,35]. Our group has recently shown that melatonin improved outcomes of renal dysfunction through the modulation of mitochondrial dynamics and function in an animal model of diabesity-induced kidney injury [36]. This mechanism could not rule out the possibility that melatonin may also depend on ER stress response and its downstream signaling mechanism to limit kidney damage under conditions of obesity and associated diabetes, given that melatonin has been shown to be effective in modulating ER stress response and UPR activation in different pathological situations [37,38,39]. With respect to kidney diseases, so far, there has been only two studies, in which melatonin was able to reduce renal ER stress in human in–vivo kidney stones model [40] and in a rat model of renal warm ischemia–reperfusion [41]. However, whether melatonin can mitigate renal ER stress in a rat model of obesity and diabetes-associated kidney injury has not yet been investigated. The present study was undertaken to investigate whether melatonin is effective against ER stress-induced renal damage in ZDF rat, and whether its renoprotective effect implicates the inactivation of UPR signaling pathway. ZDF is an excellent animal model of human obesity-induced type 2 diabetes, recapitulating pathological features similar to that seen in human, including progressive insulin resistance, glucose intolerance, hyperglycemia, hyperinsulinemia, hyperlipidemia, moderate hypertension, and progressive renal injury. The ZDF rats spontaneously develop proteinuria and focal segmental glomerulosclerosis (FSGS) by 14 to 20 weeks, which ultimately lead to renal insufficiency by 22 weeks of age [36,42,43,44,45,46]. Our findings provide new insight into the beneficial effect of melatonin supplementation on ER stress-induced kidney damage under diabesity conditions.

## 2. Results

### 2.1. Effects of Melatonin on Kidney ER Stress Response

ER stress plays an important role in the development of diabesity-related kidney disease [7]. To study ER stress in the kidney of ZDF rats and the possible effect of melatonin supplementation on this situation, we assayed the expression level of the main ER stress markers (GRP78, phospho–PERK, phospho–IRE1α, and ATF6) using Western blot analysis.

**Figure 1:**
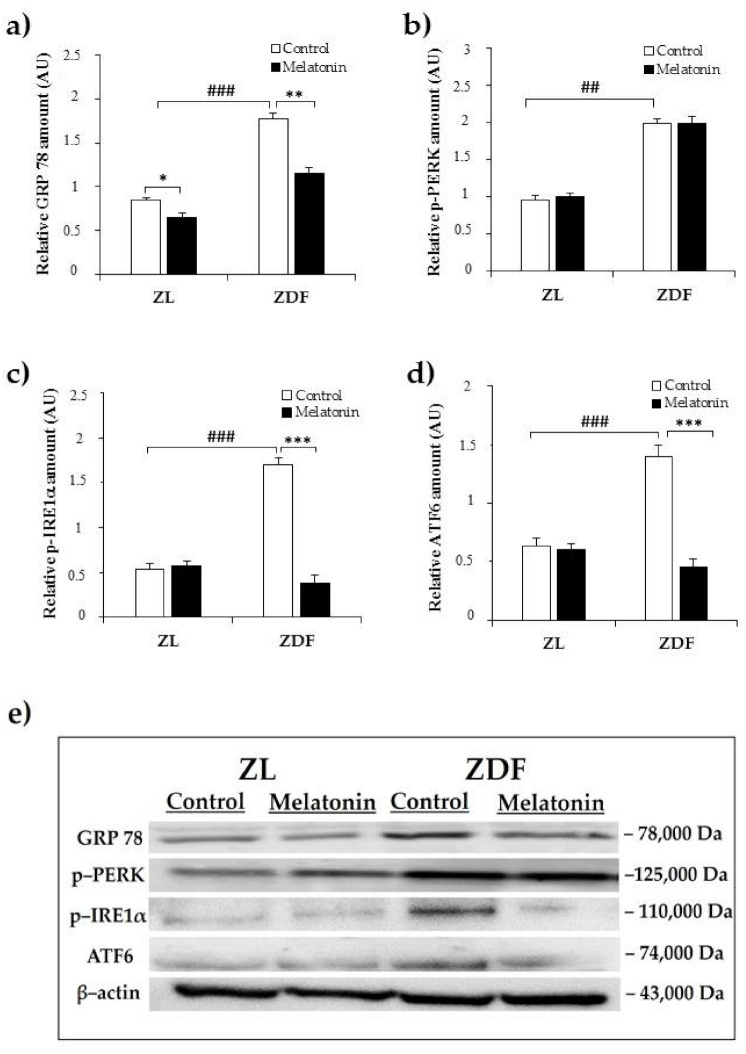
Effects of melatonin supplementation on endoplasmic reticulum (ER) stress markers in the kidney of Zücker lean and diabetic fatty rats as measured by Western blot. (**a**–**d**) Densitometry quantification of glucose-regulated protein 78 (GRP78) p–protein kinase RNA–like endoplasmic reticulum kinase (PERK), p–inositol-requiring enzyme1α (IRE1α), and ATF6 protein levels. (**e**) Representative blot of GRP78, phospho–PERK, phospho–IRE1α, and ATF6. C–ZL: control lean rats without melatonin; M–ZL: lean rats with melatonin; C–ZDF: control diabetic fatty rats without melatonin; M–ZDF: diabetic fatty rats with melatonin; ZL: Zücker lean rats; ZDF: Zücker diabetic fatty rats. Values are means ± S.E.M (*n* = 3) of ratios of specific protein levels to β–actin (Loading protein). ^##^ *p* < 0.01, ^###^ *p* < 0.001 C–ZDF vs. C–ZL; * *p* < 0.05 M–ZL vs. C–ZL; ** *p* < 0.01, *** *p* < 0.001 M–ZDF vs. C–ZDF (Tukey post hoc test).

The upregulated expression of GRP78 is considered a major hallmark of ER stress. The protein expression of GRP78 was significantly higher in the C–ZDF group compared with the C–ZL group (2.2-fold; *p* < 0.001; Figure 1a) and was found to be significantly decreased after melatonin supplementation in both ZDF (2.2-fold) and ZL (1.3-fold) groups, as compared to their control counterparts without supplementation (*p* < 0.01 and *p* < 0.05, respectively; Figure 1a).

In this study phospho–PERK, phospho–IRE1α, and ATF6 were investigated as markers of ER stress. Notably, although, the relative protein level of phospho–PERK was significantly increased in C–ZFD group compared with C–ZL group (2.1-fold; *p* < 0.01; Figure 1b), the extent of this change was not attenuated in either ZDF or ZL groups with melatonin supplementation, as compared to their corresponding without supplementation (*p* > 0.05; Figure 1b). The relative protein amount of phospho–IRE1α and ATF6 were found to be significantly increased in the C–ZDF group compared with the C–ZL group (3.1-fold and 3.0-fold, respectively; *p* < 0.001; Figure 1c,d), and melatonin supplementation lowered their expression in ZDF group (4.5-fold and 4.1-fold, respectively) but not in ZL group, as compared with the respective control ZDF (*p* < 0.001 and *p* < 0.001, respectively) and ZL (*p* > 0.05 and *p* > 0.05, respectively) without supplementation (Figure 1c,d). Interestingly, the expressed amount of phospho–IRE1α and ATF6 in M–ZDF group was restored to that of C–ZL group.

### 2.2. Effects of Melatonin on Kidney ER Stress-Related Apopotosis Markers

Apoptosis could be stimulated with the activation of prolonged UPR. To investigate whether the relieve effect of melatonin for the ER stress can be accompanied by the subsequent repression of the ER stress-induced apoptosis, we choose to evaluate the IRE1α branch of ER stress-associated apoptosis pathway.

The IRE1α contributes to the ER stress-induced apoptosis through phosphorylation of JNK, which has been proposed to be a pro-apoptotic event through down–regulation of the anti-apoptotic protein Bcl-2 and upregulation of mitochondrial-associated pro-apoptotic factors, including Bax, leading to cleavage of caspase-3, as the terminal apoptotic effector [47].

**Figure 2:**
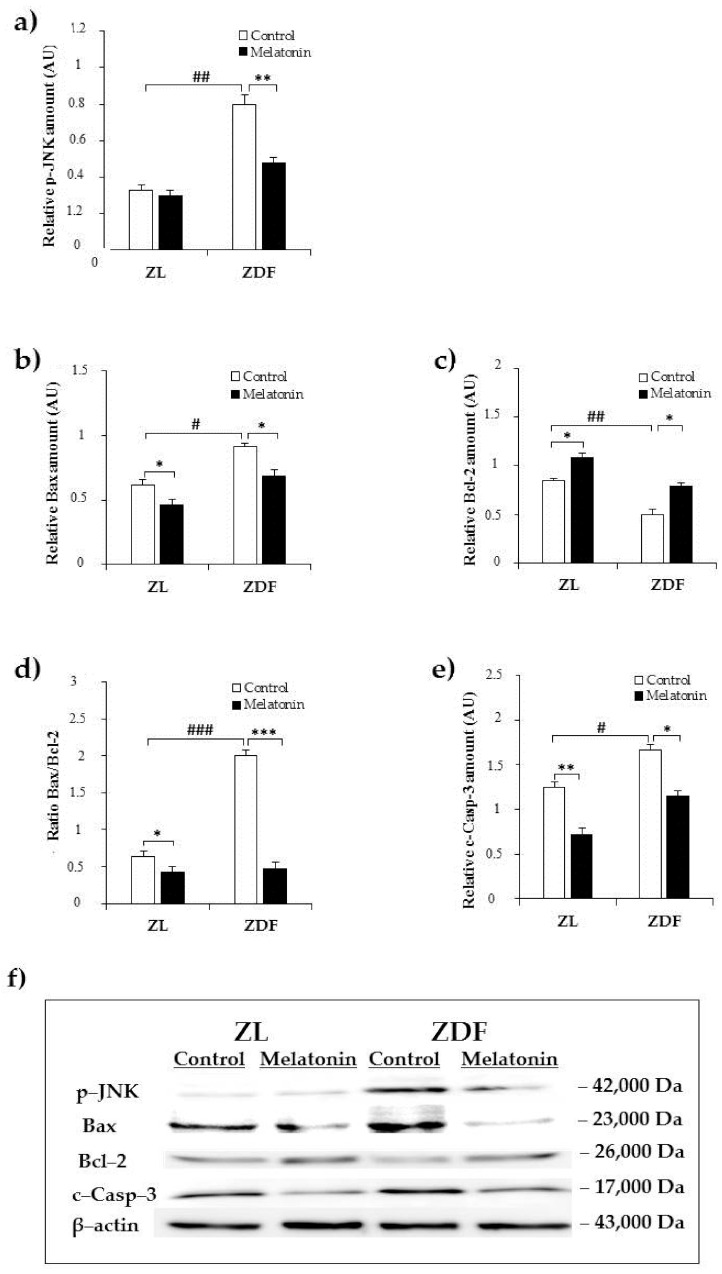
Effects of melatonin supplementation on ER stress-related apoptotic markers in the kidney of Zücker lean and diabetic fatty rats as measured by Western blot. (**a**–**c**,**e**) Densitometry quantification of p–c–jun amino terminal kinase (JNK), Bax, B cell lymphoma (Bcl)-2, and cleaved caspase-3 protein levels. (**d**) Bax/Bcl-2 ratio expression. (**f**) Representative blot of phospho–JNK, Bax, Bcl-2, and cleaved caspase-3. C–ZL: control lean rats without melatonin; M–ZL: lean rats with melatonin; C–ZDF: control diabetic fatty rats without melatonin; M–ZDF: diabetic fatty rats with melatonin; ZL: Zücker lean rats; ZDF: Zücker diabetic fatty rats. Values are means ± S.E.M (*n* = 3) of ratios of specific protein levels to β–actin (Loading protein). ^#^ *p* < 0.05, ^##^ *p* < 0.01, ^###^ *p* < 0.001 C–ZDF vs. C–ZL; * *p* < 0.05, ** *p* < 0.01 M–ZL vs. C–ZL; *** *p* < 0.001 M–ZL vs. C–ZL; * *p* < 0.05, *** *p* < 0.001 M–ZDF vs. C–ZDF (Tukey post hoc test).

We first examined the expression of the activated form of JNK (phospho–JNK). As shown in Figure 2a, western blot analysis revealed higher relative content in renal tissue from C–ZDF in comparison with C–ZL group (2.4-fold; *p* < 0.01; Figure 2a). Melatonin supplementation resulted in a significant loss of JNK phosphorylation in the ZDF group (1.7-fold) but had no effect on JNK phosphorylation in ZL group, as compared to the respective control group without supplementation (*p* < 0.01 and *p* > 0.05, respectively; Figure 2a). In line with this, we also evaluated the expression of mitochondria-associated pro-apoptotic Bax and anti-apoptotic Bcl-2 factors. The analysis showed that protein expression of Bax significantly increased in C–ZDF group when compared to C–ZL group (1.6-fold; *p* < 0.05; Figure 2b). This was concomitant with a significant decrease in the protein level of Bcl-2 in the C–ZDF group, as compared to C–ZL group (1.9-fold; *p* < 0.01; Figure 2c). These protein changes resulted in a significant increase in Bax/Bcl-2 ratio, as compared to C–ZL group (3.1-fold; *p* < 0.001; Figure 2d). After melatonin supplementation, the protein level of Bax significantly reduced, whereas that of Bcl-2 increased, resulting in a reduced Bax/Bcl-2 ratio expression in both ZDF (1.5-fold, 2.9-fold, and 4.2-fold, respectively) and ZL (1.3-fold, 1.3-fold, and 1.5-fold) groups, as compared to the corresponding control ZDF (*p* < 0.05, *p* < 0.05, and *p* < 0.001, respectively) and ZL (*p* < 0.05, *p* < 0.05, and *p* < 0.05, respectively) groups without supplementation (Figure 2b–d). To finally explore the relevance of melatonin on ER stress-mediated apoptosis, we detected the protein level of the activated form of caspase-3. We found that the level of activated caspase-3 (cleaved caspase-3) was significantly higher in C–ZDF group than in C–ZL group (1.4-fold; *p* < 0.05; Figure 2e), and that melatonin supplementation significantly reduced its cleavage in both ZDF (1.8-fold) and ZL (1.7-fold) groups compared to their control counterparts without supplementation (*p* < 0.05 and *p* < 0.01, respectively; Figure 2e). Interestingly, the expression levels of all the aforementioned ER stress-related apoptotic markers (phospho–JNK, Bax, Bcl-2, and cleaved caspase-3) in M–ZDF group were restored to those of the C–ZL group.

### 2.3. Effects of Melatonin on Renal Tissu Morphology

**Figure 3:**
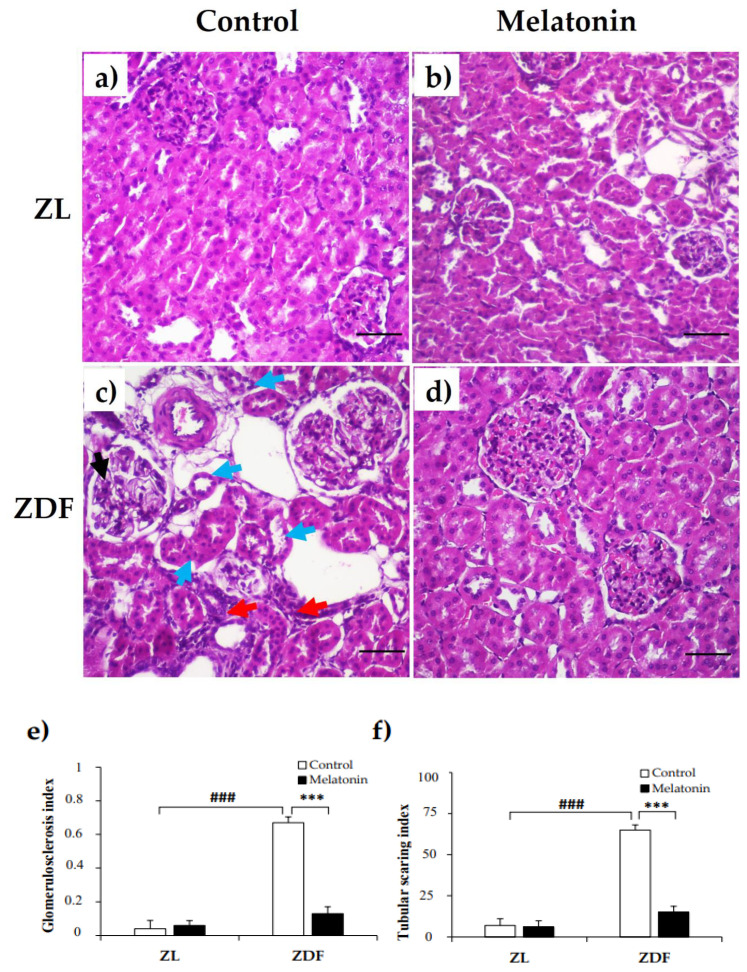
Effects of melatonin on renal tissue morphology of Zücker lean and diabetic fatty rats. (**a**,**b**) Representative photomicrographs showing normal appearance of the glomerulus and tubules in C–ZL (left panel) and M–ZL rats (right panel). (**c**) Early focal segmental glomerulosclerosis (FSGS) and tubulointerstitial damage were noted in C–ZDF rats, with a left glomerulus showing segmental mesangial hypercellularity and mild segmental endocapillary (black arrow) and on the right showing hypertrophy, associated with interstitial fibrosis (red arrows) and mild tubular degeneration as shown with epithelial cells atrophy and loss of brush borders and cell polarity (blue arrows). (**d**) Normal structure of renal tissue was observed in M–ZDF rats, with lower severity of FSGS and normal renal tubules, and no signs of fibrosis. (H&E stain; original magnification at ×100). (**e**,**f**) Semi-quantitative analysis of glomerulosclerosis index (left panel) and tubular index (right panel) scores in ZL and ZDF rats. C–ZL: control lean rats without melatonin; M–ZL: lean rats with melatonin; C–ZDF: control diabetic fatty rats without melatonin; M–ZDF: diabetic fatty rats with melatonin; ZL: Zücker lean rats; ZDF: Zücker diabetic fatty rats. Values are means ± S.E.M (*n* = 6). ^###^ *p* < 0.001 C–ZDF vs. C–ZL; *** *p* < 0.001 M–ZDF vs. C–ZDF (Tukey post hoc test). Scale bar: 200 µm.

Given our previous findings of improved renal dysfunction outcomes with melatonin supplementation [36], we next examined whether melatonin can prevent renal structural damage. Compared with renal tissues from C–ZL rats, C–ZDF rats exhibited an early FSGS with glomerular hypertrophy, mesangial expansion, segmental mesangial hypercellularity, and capillary collapse (endocapillary) (Figure 3c). Furthermore, tubulointerstitial damage, such as interstitial fibrosis and dilated tubules with atrophic epithelial cells and destructed brush borders were also observed in C–ZDF rats (Figure 3c). The semi-quantitative histopathological analysis indicated that both glomerulosclerosis index and tubular scarring index scores were significantly higher in C–ZDF rats compared with C–ZL rats (0.67 ± 0.03 vs. 0.04 ± 0.05/100 glomeruli and 65.42 ± 3.12 vs. 7.12 ± 4.11/100 glomeruli, respectively; *p* < 0.001; Figure 3e,f). Melatonin supplementation prevented FSGS and tubular degeneration (Figure 3d) and significantly lowered the glomerulosclerosis index and tubular index scores in ZDF rats (0.23 ± 0.04/100 glomeruli and 11.78 ± 3.71/100 glomeruli, respectively), as compared to their control counterparts without supplementation (*p* < 0.001; Figure 3e,f). The renal tissue from M–ZL rats exhibited a similar histological aspect to that from C–ZL rats (Figure 3a,b), and no significant difference in either glomerulosclerosis index or tubular scarring index was observed in them (0.06 ± 0.03/100 glomeruli and 6.23 ± 3.89/100 glomeruli, respectively), when compared to their respective control rats without supplementation (*p* < 0.001; Figure 3e,f).

## 3. Discussion

Results from this study revealed for the first time that melatonin supplementation alleviated renal ER stress response and subsequent pro-apoptotic IRE1α–JNK signaling pathway in a rat model of diabesity-induced kidney injury. This was associated with the restoration of normal renal morphology. Based on these findings, we suggest that the therapeutic benefit of melatonin in the obese and diabetic kidney could be attributed, at least in part, to the modulation of ER stress-mediated cell death pathway. The present study also stresses the role of renal ER stress in the pathogenesis of diabesity-related kidney injury

ER stress has been reported to be activated in renal tissue under conditions of obesity and associated diabetes, contributing to the development and progression of kidney disease [48]. In the present study, we proved that ER stress was induced in the renal tissue from control diabetic obese rats, as evidenced by amplified levels of GRP78, phospho–PERK phospho–IRE1α, and ATF6 stress markers. The precise trigger of ER stress cannot be deduced from this study. However, we postulate that it is likely to be a combination of increased reactive oxygen species, hyperglycemia, lipid accumulation, and excessive protein load in the ER, known to occur under obese and diabetic conditions [3]. Interestingly, the current study showed that melatonin supplementation usefully restored the levels of GRP78, phospho–IRE1α, and ATF6 proteins in diabetic obese rats. Meanwhile, melatonin supplementation had no effect on the protein level of phospho–PERK. These data clearly indicate that melatonin may exert a protective effect on obese diabetic kidneys by inhibiting the IRE1α and ATF6 pathways. Our results are in keeping with recent findings demonstrating that melatonin reduced ER stress in different models of kidney injury. For instance, melatonin attenuated the ER stress response markers in–vitro kidney stones model [40]. Similar effects were also observed in rat model of renal ischemia/reperfusion injury [41]. In lean rats, melatonin supplementation reduced the levels of GRP78 but had no effect on the three ER stress sensors, suggesting reduced acute ER stress level, which possibly reflects the fact that melatonin minimizes oxidative stress that is known to cause accumulation of unfolded proteins; bearing additionally in mind that myriad of studies, including ours, highlighted melatonin as a powerful antioxidant [16,49]. To our knowledge, this study provides the first evidence that melatonin might exert its protective effects on obese diabetic kidneys through inhibiting ER stress.

Based on the above finding showing that melatonin preferentially suppressed the IRE1α and ATF6 pathways among the three major arms of the ER stress response, we investigated the effect of melatonin on the protein expression of some IRE1α-downstream targets. It is known that IRE1α branch played a vital role in ER stress related to apoptosis [47]. This branch is highly regulated and could be the link between the survival role of ER stress and the apoptotic features related to UPR [50]. During prolonged ER stress, enhanced IRE1α kinase activity can phosphorylate the downstream JNK target to induce cell death. The induction of JNK is regarded as an important element of the switch from pro-survival to pro-apoptotic signaling cascades [47]. This study clearly shows that renal tissue from control diabetic obese rats contain a high amount of phosphorylated JNK, which indicates the activation of the apoptotic signaling. The activation of JNK signaling is critical in the development of various forms of human kidney injury [51]. JNK activation was also been evident in wide array of animal models of glomerular diseases and in the aging kidney [52,53,54,55,56,57]. ER stress-associated apoptosis is a complex process controlled by many factors, such as Bax and Bcl-2. Bcl-2 is an anti-apoptotic factor that reduces the permeability of the mitochondria membrane and regulates the mitochondrial apoptosis, while Bax is a pro-apoptotic factor that promotes the activation of caspase-3 [47]. It has been reported that the phosphorylation of IRE1α/JNK activates both pro-apoptotic Bax and concomitantly inhibited Bcl-2 factor [47]. Consistent with the activated IRE1α pro-apoptotic pathway in control diabetic obese rats, we logically found an increased level of apoptotic cell markers (Bax and cleaved caspase-3) and decreased level of anti-apoptotic Bcl-2 protein in the control diabetic obese rats compared with control lean rats. The ratio of Bax/Bcl-2 is regarded as a key factor in determining whether cells can enter the apoptosis process [58]. Our data showed an elevated Bax/Bcl-2 ratio, which confirms the activation of the apoptotic pathway. Apoptosis is considered to be closely associated with the pathogenesis of diabetic and obesity-related kidney disease [59]. It has also been reported that diabetic and obesity-related kidney disease undergoes apoptosis in response to diabetic hyperglycemia and proteinuria [6].

In the current study, melatonin supplementation attenuates the upregulation of JNK and Bax expressions, the cleavage of caspase-3, the downregulation of Bcl-2 expression, and the Bax/Bcl-2 ratio expression in renal tissue from diabetic obese rats. Based on these data, we speculate that melatonin protects against kidney damage occurred under obese and diabetic state, by activating the pro–survival mechanisms and preventing the excessive upregulation of pro-apoptotic pathways. Moreover, because melatonin completely eliminated the induction of JNK signaling effectors and restored them to those of the control lean rats, we hypothesize that melatonin intervened with the downstream components of the JNK signaling pathway rather than it controls upstream of the ER stress signaling. Results from lean rats are a further argument in support of this hypothesis, according to which melatonin enhanced the level of Bcl-2 and reduced that of Bax and cleaved-caspase-3 but had no effect on the level of either JNK or IRE1α, which would, on the one hand, be in a favor of adaptive capacity enhancement and, on the other, be explained by the fact that melatonin target components of the Bcl-2 signaling in order to suppress the IRE1α/JNK pro-apoptotic pathway, which remains to be verified. In support with this suggestion, melatonin was shown to interfere with the intrinsic pathway of apoptosis by inducing the mitochondrial re–localization of Bcl-2 [60] and directly inhibiting Bax and caspase-3 activation [61]. Previously, other examples of melatonin reducing the ER stress-associated apoptotic conditions have been reported. For instance, melatonin protected the heart by ameliorating cardiac ER stress-induced apoptosis in rat with diabetic cardiomyopathy [62]. Additionally, melatonin protected the brain against ischemia–reperfusion injury by attenuating ER stress trigged autophagy through both PERK and IRE1α pathways [63]. Inhibition of the JNK pathway by genetic or pharmacologic approaches has also been demonstrated to be effective at preventing and suppressing glomerulosclerosis and tubulointerstitial fibrosis [51]. The current data demonstrated that in addition to suppression of the IRE1α branch of JNK signaling, melatonin supplementation prevented glomerulosclerosis, glomerular hypertrophy, and tubulointerstitial damage, which are common features seen in patients with obesity and T2DM [64,65]. Of note, a positive correlation between elevated JNK activation and increased glomerulosclerosis was observed in human renal biopsies from patients with hypertension and diabetic nephropathy [66]. Taken together, the present data suggest that melatonin might exert its renoprotective effect by targeting components of the pro-apoptotic IRE1α/JNK pathway.

Even though our current and previous data suggest that the renoprotective mechanism of melatonin under conditions of obesity and diabetes may be due, partially, to the suppression of ER stress and related IRE1α branch of JNK signaling and modulation of the mitochondrial fission/fusion balance and function, it could not rule out the possibility that the protective effect of melatonin on kidney may also be attributed to the improvement of the metabolic complications of obesity, such as dyslipidemia, insulin resistance, hyperglycemia, hypertension, and oxidative stress, which are well accepted as risk factors for the development of kidney disease [67], given that several studies, including ours, showed a beneficial effect of melatonin to counteract these risk factors [49,68,69].

## 4. Materials and Methods

### 4.1. Reagents

All reagents used were of the highest purity available. Melatonin was purchased from Sigma-Aldrich (Madrid, Spain).

### 4.2. Animals and Experimental Protocol

Male Zücker diabetic fatty rats (ZDF; *fa*/*fa*) and their male lean littermates (ZL; *fa*/–) were purchased at 5 weeks of age from Charles River Laboratory (Charles River Laboratories, SA, Barcelona, Spain). This study was conducted in compliance with the European Union guidelines for animal care and protection. Rats were housed 2 per clear plastic cage under a 12–h light/dark cycle (lights on at 07:00 a.m.) in a temperature-controlled room (25–28 °C). Tap water and Purina 5008 rat chow (protein 23%, fat 6.5%, carbohydrates 58.5%, fiber 4%, and ash 8%; Charles River Laboratories, SA, Barcelona, Spain) were provided *ad libitum*. All animals were acclimatized for 1 week before starting the experiments.

At the age of 6 weeks, the animals were randomly divided into four groups (*n = 6* per group): the non-supplemented control groups (C–ZDF and C–ZL) and the melatonin–supplemented groups (M–ZDF and M–ZL). Melatonin was dissolved in a minimum volume of absolute ethanol and then diluted to the final solution of 0.066% (*w/v*) in the drinking water to yield a dose of 10 mg/kg body weight and was received daily for 17 weeks. The animals in the non-supplemented control groups received the vehicle in the drinking water at a comparable dose and supplementation duration. Fresh melatonin and vehicle solutions were prepared twice a week, and the melatonin dose was adjusted for body weight over the entire period of the study. Water bottles were covered with aluminum foil to protect from light, and the drinking fluid was changed twice weekly.

At the end of the experiment, the animals were sacrificed under sodium thiobarbital (thiopental) anesthesia, and the kidney of each rat was immediately removed and frozen at −80 °C until further use.

### 4.3. Protein Extraction and Western Blotting

Western blot analysis was performed according to the instructions previously described by our research group [70]. Briefly, about 200 mg of kidney tissue was homogenized in lysis buffer (150 mM NaCl, 5 mM ethylene diamine tetra-acetic acid EDTA, 50 mM Tris–HCl; pH 7.4) without Triton X–100 and homogenized with a Teflon pestle, maintaining the temperature at 4 °C throughout all procedures. Homogenates were centrifuged (3000× *g* × 15 min; 4 °C), and the fat cake was removed from the top of the tube. Then, Triton X–100 was added to a final concentration of 1%. After incubating at 4 °C for 30 min, extracts were cleared by centrifugation at 15,000× *g* for 15 min at 4 °C. The supernatant fraction was stored at 80 °C in aliquots until use. Protein concentration was measured by the Bradford method using the bovine–serum albumin (BSA) as a standard. Equal amounts of protein extracts were resolved on SDS–PAGE (sodium dodecylsulfate polyacrylamide gel electrophoresis). The gels for immunoblot analyses were transferred to a nitrocellulose membrane (Bio-Rad Trans-Blot SD, Bio-Rad Laboratories). The membranes were then blocked with 5% non-fat dry milk in tris-buffered saline (TBS) containing 0.05% Tween-20 (TBS-T) for 1 h at 37 °C and incubated overnight at 4 °C with primary antibodies against GRP78 (cat#G–8918), phospho–PERK (cat#SAB–5700521), phospho–IRE1α (cat#I–6785), ATF6 (cat#SAB–2100170), phospho–JNK (cat#07–175), Bax (cat#SAB–4502546), Bcl-2 (cat#SAB–4500003), and cleaved caspase-3 (cat#AB–3623). All antibodies were obtained from Sigma-Aldrich (Sigma-Aldrich, Madrid, Spain) at 1:200–1:2000 dilution with TBS–T containing 2.5% non-fat dry milk. Equal loading of protein was demonstrated by incubating the membranes with mouse β–actin antibody (cat#SC–81178) (Santa Cruz Biotechnology, Santa Cruz, CA, USA) at 0:1000 dilution. After the incubation, the membranes were washed three times for 20 min in TBS-T and incubated for 1 h at room temperature with respective horseradish peroxidase–conjugated secondary antibodies (Sigma-Aldrich, Madrid, Spain) at 1:1000 dilution. The membrane was washed three times for 20 min in the TBS-T, and then a chemiluminescence assay system (ECL kit, GE Healthcare Life Sciences, Buckinghamshire, UK) was used to develop the immunoreactive bands. Finally, the protein band densities were quantitatively analyzed using Image J 1.33 software (National Institutes of Health, Bethesda, MD, USA). The results were normalized to β-actin as a loading control. All experiments were performed in triplicate.

### 4.4. Microscopic Analysis

Kidney tissues were cut into sections and fixed with 4% paraformaldehyde for 24 h, dehydrated, and embedded in paraffin following routine protocols. After embedding in paraffin, 4-μm-thick sections were stained with hematoxylin and eosin (H&E) and finally inspected under a light microscope (Olympus, Germany) equipped with a digital camera system (Carl Zeiss camera, model Axiocam ERc 5s. Göttingen, Germany). The glomerular lesions and tubular degeneration were evaluated semiquantitatively. The glomerular lesion was defined as glomerular hypertrophy, mesangial expansion, glomerulosclerosis, and capillary occlusion, and semiquantitatively scored from 0 to 3 + on the basis of severity of the lesion for each glomerulus (0 = none, 1 < 25%, 2 = up to 50%, and 3 > 50%). The glomerulosclerosis index was then calculated as described [45]. Briefly, 100 glomeruli were randomly chosen from each rat kidney and carefully scored for glomerular lesion and then adding all the scores and divided by 100. This was done in a sequential manner to ensure that the same glomerulus was not graded twice. To evaluate tubular degeneration, tubular scarring index was evaluated by counting the total number of atrophic or atrophying tubular in the same area that contained these 100 glomeruli in each section [45].

### 4.5. Statistical Analysis

Statistical Package of Social Science (IBM SPSS Software, version 15, Michigan, IL, USA) was used for statistical analysis. All results are expressed as mean ± standard error of the mean (S.E.M) values. Comparisons between experimental groups were analyzed using one-way ANOVA followed by Tukey post hoc test. Differences between group means were considered statistically significant if *p* < 0.05. A *p* < 0.05 was considered statistically significant, and levels of significance were labeled on the figures as follows: *** *p* < 0.001; ** *p* < 0.01; * *p* < 0.05, and ^###^ *p* < 0.001; ^##^ *p* < 0.01; ^#^ *p* < 0.05.

## 5. Conclusions

This study is the first to demonstrate that chronic melatonin supplementation in obese and diabetic-induced kidney injury rat model acts as a renal ER stress suppressor, and the mechanism is possibly through targeting IRE1α signaling pathway. This, in concert with our previously reported effect via mitochondria in the same strain animal model, showing that melatonin supplementation improves kidney function under conditions of obesity and diabetes [34]. Data from our unpublished studies showed that melatonin doses ranged between 1–10 mg/kg body weight (BW) reduces body weight and improves metabolic outcomes in diabetic obese rats. Based on human equivalent dose calculation [71], we estimated that a clinically effective dose of 0.16–1.6 mg/Kg BW might have potential therapeutic implications, especially among the obese diabetic population with a high risk of kidney disease. Hence, clinical trials should be promoted to investigate the optimal effective dose in humans. Further in–vitro studies are required to fully elaborate on the effect of melatonin on ER stress-induced kidney injury and decipher the precise underlying related molecular mechanism.

## Data Availability

Not applicable.
